# Unveiling the Genomic Architecture of Phenotypic Plasticity Using Multiple GWAS Approaches Under Contrasting Conditions of Water Availability: A Model for Barley

**DOI:** 10.3390/ijms27020652

**Published:** 2026-01-08

**Authors:** Sebastián Arenas, Andrés J. Cortés

**Affiliations:** 1 Department of Plant Protection Biology, Swedish University of Agricultural Sciences, 23422 Lomma, Sweden; 2 Facultad de Ciencias Agrarias—Departamento de Ciencias Forestales, Universidad Nacional de Colombia—Sede Medellín, Medellín 050034, Colombia

**Keywords:** abiotic stress tolerance, drought stress, genomic bases of plasticity, genetic mapping, GWAS, antagonistic pleiotropy, *Hordeum vulgare*

## Abstract

Phenotypic plasticity is a key mechanism by which crops adjust to fluctuating environmental conditions, yet its genetic basis under drought remains poorly characterized in barley (*Hordeum vulgare*). We hypothesized that phenotypic plasticity under drought is controlled by a distinct, trait-specific genetic architecture that can be detected using complementary plasticity metrics and genome-wide association studies (GWAS). Here, we examined data from 1277 spring barley genotypes grown under well-watered and water-limited conditions to quantify plastic responses across two developmental traits (i.e., heading time, and maturity) and seven productivity-related traits (i.e., total dry matter, plant grain yield, grain number, grain weight, harvest index, vegetative dry weight, and grain-filling period). The experimental design, based on contrasting water regimes across a large diversity panel, allowed robust assessment of genotype-by-environment interactions. We combined five complementary plasticity estimators with four independent GWAS approaches to resolve the genomic architecture underlying trait-specific plasticity. Environmental effects dominated variation in yield-related traits, whereas developmental traits remained more genetically determined. The different plasticity metrics captured distinct but partially overlapping response dimensions, and their integration greatly increased the robustness of association signals. A total of 239 high-confidence SNPs obtained for top traits, those associated across metrics and methods, were enriched in coding regions and mapped to genes involved in osmoregulation, carbohydrate metabolism, hormonal pathways, and ion transport. A total of 27 high-confidence SNPs were located in coding regions, showing genotype-specific differences in the magnitude and even direction of phenotypic plasticity. These loci exhibited opposite allelic effects across water regimes, consistent with context-dependent antagonistic pleiotropy. The fact that candidate alleles for the plastic response modulate environmental sensitivity differently highlights that drought resilience arises from environment-contingent genetic architectures. Overall, these results provide a comprehensive framework for dissecting plasticity and identify concrete genomic targets for indirect selection targeting crop resilience with improved performance under increasingly variable water availability.

## 1. Introduction

Water availability is one of the major environmental constraints limiting agricultural productivity worldwide [1]. Increasingly frequent and intense drought events, driven by ongoing climate change, substantially reduce crop performance and yield stability, especially in semi-arid regions where water scarcity strongly restricts plant growth, reproduction, and survival [2]. Therefore, understanding the underlying mechanisms on how plants perceive, respond, and adapt to fluctuating water conditions is therefore essential for maintaining crop resilience and ensuring future food security [3,4,5].

Barley (*Hordeum vulgare* L.) is a globally important cereal cultivated across a broad range of agro-ecological zones, from temperate to dryland environments [6]. Its wide environmental breadth and agronomic relevance make it an excellent model for dissecting the mechanisms responsible for plant plasticity and adaptation to abiotic stress, including drought and water limitation [7]. Despite this, our understanding of the physiological and genetic processes governing barley’s responses to contrasting water regimes remains incomplete, particularly regarding the genetic determinants of phenotypic plasticity [8].

Plant responses to water limitation arise from a suite of avoidance, tolerance, and resilience mechanisms that operate across biological scales [9], from gene expression and metabolic adjustments to morphological changes and final yield components [10]. Many of these responses are mediated by phenotypic plasticity, classically defined as the capacity of a genotype to alter its phenotype in response to environmental variation [11,12]. Plasticity, together with genetic adaptation, are widely recognized as key drivers of plant responses to heterogeneous environments [13]. Yet, although the ecological relevance of plastic responses is well established, its genetic architecture remains poorly characterized, often assuming that it is merely a secondary consequence of stress tolerance. Instead, plasticity is starting to be recognized as a genetically regulated trait that varies among genotypes within the same species [14,15]. Accurately quantifying plasticity is therefore essential for identifying genes and pathways that control plastic plant responses under climate change.

A variety of methodological approaches have been proposed to quantify plasticity and genotype-by-environment interactions (G×E) [14,16]. Simple indices, such as trait ratios, absolute differences, or covariances between environments, capture broad phenotypic shifts, while more integrative estimators such as the Relative Distance Plasticity Index (RDPI), reaction-norm slopes derived from linear models, and multivariate approaches capture distinct dimensions of plastic behavior, including magnitude, direction, and stability. Because these estimators are not equivalent and emphasize different biological interpretations, combining them provides a more complete picture of the plastic response [17].

Meanwhile, genome-wide association studies (GWAS) offer a powerful framework for identifying loci underlying plasticity (as per a specific estimator) [18,19,20,21]. However, classical GWAS models face well-known limitations [22,23,24], including reduced power for complex traits [20,25], sensitivity to population structure [15,26,27], and difficulty capturing non-additive or interactive genetic effects [28,29,30]. This being said, merging multiple GWAS approaches has emerged as an effective strategy for detecting robust and reproducible associations [18,19]. Mixed-model approaches (e.g., rrBLUP, BLINK) provide statistical rigor and account for population stratification and kinship, whereas machine learning methods (e.g., Random Forest, XGBoost) capture non-linear patterns and higher-order interactions often missed by linear models. Overlapping signals across methods would substantially increase confidence in candidate loci and reduce model-specific false positives. Applying integrative GWAS framework to phenotypic plasticity [20,31] under contrasting water regimes offers a unique opportunity to uncover the genetic basis of drought-related plastic responses [32,33]. Genomic regions consistent across plasticity metrics and methodological approaches are likely to represent key components of drought response pathways that modulate performance and stability in variable environments [34,35,36,37].

Therefore, the main objective of this study was to dissect the genomic architecture of phenotypic plasticity in a diverse panel of 1277 barley genotypes grown under well-watered and water-limited conditions [8]. After integrating multiple plasticity estimators with complementary GWAS methodologies, we aim to: (i) identify loci and candidate genes associated with plastic responses to drought, (ii) explore whether they differ in direction and magnitude, (iii) characterize the biological processes underlying adaptive phenotypic shifts, and (iv) demonstrate the value of utilizing a diversity of plasticity estimators and GWAS methods in resolving complex genetic architectures.

We hypothesize that alleles that enhance barley performance under drought impose a trade-off on fitness cost under well-watered conditions due to antagonistic pleiotropy acting on shared metabolic and developmental pathways [16,38]. This work provides an integrative framework for linking plasticity metrics, genomic variation, and functional annotation, ultimately contributing to a deeper understanding of how barley, and potentially other cereals, modulates its phenotype under fluctuating water environments. Our findings offer insights relevant for breeding programs focused on enhancing drought resilience through a more comprehensive understanding of phenotypic plasticity.

## 2. Results

### 2.1. Phenotypic Responses to Water Availability

Phenotypic summary statistics analyses confirmed that the contrast between well-watered (WW) and water-limited (WL) treatments produced significant differences for six evaluated traits (Table 1), which were retain herein after.

**Figure 1:**
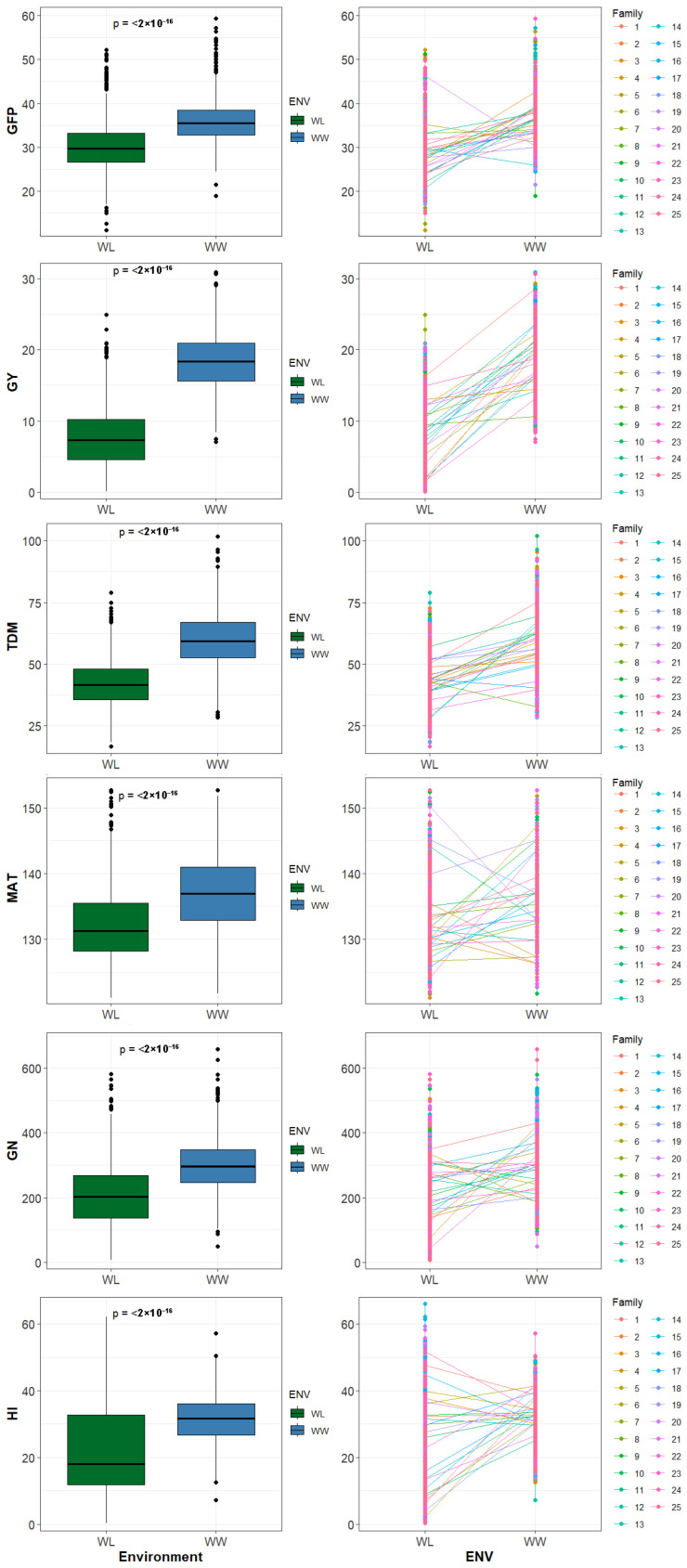
Phenotypic variation across water regimes. Boxplots (**left**) and line plots (**right**) show the distribution of traits in barley grown under well-watered (WW) and water-limited (WL) conditions. Each point represents an individual genotype. Wilcoxon rank-sum tests reveal significant differences between environments (*p* < 0.001). Trait abbreviations are detailed in Table 1.

Genotypes presented consistently higher values under WW conditions, a pattern corroborated by highly significant Wilcoxon tests (Figure 1). Marked reductions were observed under WL in productive variables, especially in GY, GN, DM, and HI. In contrast, developmental traits such as HEA and MAT showed more stable responses, with less variation between treatments. Line plots showed slight heterogeneity in response: while most showed a decrease in yield and biomass under WL, a subset of lines showed opposite patterns, with increases in value under stress conditions. This divergent behavior was evident in the reaction norms (Figure S1), where genotype–environment trajectories revealed that phenotypic plasticity varies not only in magnitude but also in direction.

### 2.2. Variance Partitioning and G×E Interactions

The variance decomposition (Table 2) revealed that the relative importance of genetic (G) and environmental (E) components varied considerably between traits. The results showed a dependence on the environmental context. In the case of GY, the environment explained around 80% of the variation, while the VG contributed only 7%, indicating a strong dependence of performance on water availability. In contrast, in HEA traits, the genetic component accounted for more than 70% of the variance, with a reduced environmental effect, reflecting stricter and more stable genetic control under stress conditions. Other traits such as GFP, GN, HI, TDM, and MAT showed intermediate proportions, suggesting a complex interaction between genotype and environment (G×E). The GW trait was discarded because the proportion of VG explained was not significant, while HEA was excluded due to the absence of a relevant VE effect on water limitation. Likewise, VDW was discarded because it was the trait with the highest proportion of variation explained by residual variance. These variance patterns highlight the diversity of mechanisms underlying different types of traits, from those strongly determined by genetics to those more modulated by the environment. Thus, six traits were retained for further analysis (GFP, GN, GY, TDM, MAT, and HI, as abbreviated in Table 1).

The comparison of phenotypic means under contrasting irrigation conditions for the six selected traits (Table 1) showed significant reductions in most of the traits under WL. GY decreased from 18.33 g in WW to 7.63 g in WL, representing a reduction of nearly 60%. Similarly, GN decreased from 300.3 to 207.8, demonstrating the direct influence of water availability on reproductive capacity. TDM also decreased from 59.93 g in WW to 42.17 g in WL, reflecting a generalized physiological impact. These decreases were accompanied by an increase in variability between genotypes, suggesting genetic differences in response to water deficit. HI and GFP showed intermediate responses. HI decreased from 31.6% in WW to 22.2% in WL, indicating lower efficiency in converting biomass to grain under water limitation, and GFP decreased from 36.01 to 30.09 days.

The PP estimators (Figure S2) unveiled positive correlations with each other, validating that they capture related, although not identical, facets of the response. CV was discarded due to its high correlation with other variables, leaving WW, WL, Ratio, RDPI, and Linear as the central metrics, which were used to structure the association analyses.

### 2.3. Population Structure and Linkage Disequilibrium

**Figure 2:**
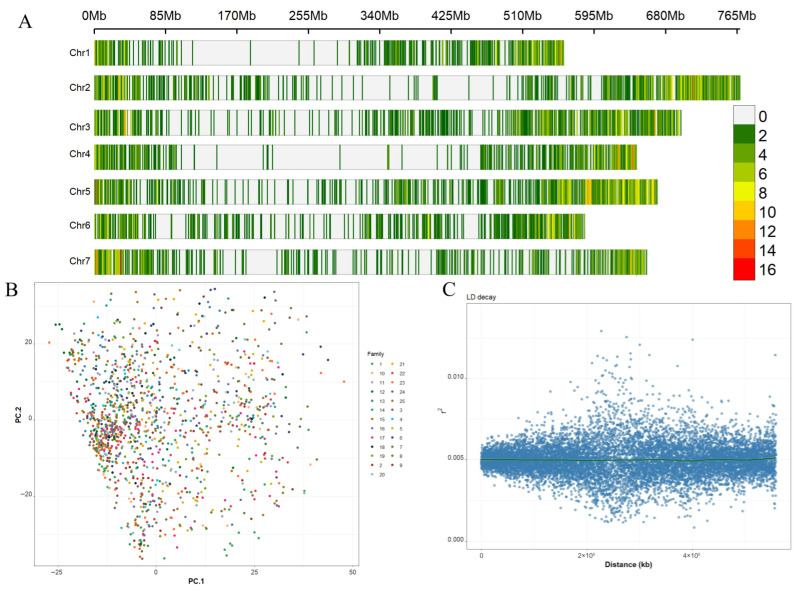
Population structure and linkage disequilibrium in the HEB population. (**A**) Genome-wide marker distribution across the seven barley chromosomes. (**B**) Principal component analysis (PCA) of 1277 barley genotypes based on genome-wide SNP data. The first two components explain major axes of population structure and separate families within the population. (**C**) Genome-wide LD decay estimated by *r*^2^ as a function of physical distance. The observed rapid LD decay indicates high mapping resolution, supporting the power of subsequent GWAS analyses.

Population structure analysis (Figure 2A) showed that the SNPs in the study are distributed randomly across barley chromosomes. It was also observed that the 1277 genotypes were distributed in undifferentiated groups, without drastic genetic barriers, and therefore the genetic structure was very weak (Figure 2B). This aligns with the neutral hypothesis of random mating and population panmixia.

Additionally, the estimation of linkage disequilibrium (LD decay) indicated a rapid loss of correlation with physical distance, ensuring high resolution power for the detection of associations at the SNP level (Figure 2C). Low genetic structure allowed effective exploration of genetic variation and the genomic architecture responsible for trait variation without compromising the statistical power of the associations.

### 2.4. Genotype–Phenotype Association (GWAS)

#### 2.4.1. Overlap Between GWAS Methods

**Figure 3:**
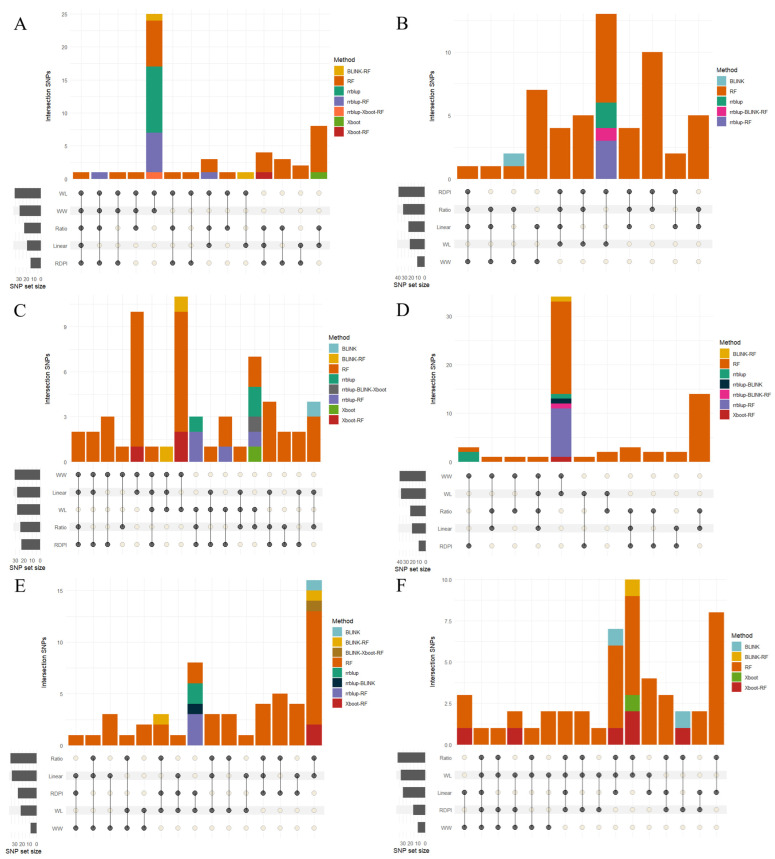
Summary of GWAS results obtained using four approaches: BLINK, rrBLUP, Random Forest (RF), and XGBoost (colored barplots) across different plasticity components (horizontal grey bars at the bottom of each subpannel). Results are presented for the traits GFP (**A**), GY (**B**), TDM (**C**), MAT (**D**), GN (**E**), and HI (**F**). Several markers were consistently detected across multiple methods, strengthening the confidence in associations. Identified loci underlying phenotypic plasticity to water stress in barley compare consistently across methodologies.

The association analyses (GWAS) performed using the four approaches (BLINK, rrBLUP, RF, and XGBoost) detected a considerable number of SNPs associated with the traits and with the different PP estimators (Figure 3).

The QTLs identified by two or more plant status estimators were considered high-priority candidates, which substantially reduced the initial list of markers and increased confidence in the retained associations, as they represented consistent and robust signals in the face of methodological differences. Overall, the bar graphs in Figure 3 summarized the distribution of significant QTLs obtained by different methods and metrics. In each panel, corresponding to the GFP, MAT, GY, GN, TDM, and HI traits, the colored bars reflected the frequency of association detection by method, while the lower dots indicate the intersections between the absolute values (WW, WL) and the plasticity metrics (Ratio, Linear, and RDPI).

Comparative analysis between traits showed notable variations in the consistency and overlap of associations between methods. The GFP and MAT traits showed the greatest overlap of SNPs, with several markers detected consistently by three or more approaches. In contrast, GY and GN showed more dispersed associations with less overlap between methods, while TDM recorded a limited number of signals without consistent overlaps. The HI trait exhibited a more defined pattern, with a recurring group of SNPs detected by rrBLUP, RF, and XGBoost.

Overall, the bar plots reflect heterogeneity in the density and overlap of associations between traits, highlighting the ability of different GWAS approaches to capture the genetic complexity underlying phenotypic plasticity under contrasting water availability conditions. It was observed that a machine learning-based method (RF) tended to identify a greater number of unique associations, while BLINK and rrBLUP concentrated on more conservative signals but coincided with other approaches. BLINK and Xboot were the most stringent methods and generated the fewest individual associations. HI was the only one that presented common associations for three different methodologies. The highest intersection sets corresponded to combinations of rrBLUP-RF and BLINK–rrBLUP, which reinforces the complementarity of statistical in the detection of relevant loci.

#### 2.4.2. Trait-Specific Patterns of Association

PP estimators showed variable patterns between traits and methods, revealing both common and specific metrics. In general, the WW and WL indices concentrated the highest number of associations, followed by derived estimators such as Ratio and Linear, while RDPI showed a lower detection frequency. In the GFP and MAT traits, repeated associations were observed between multiple metrics, indicating a broad coincidence of SNPs detected under different plasticity approaches. In contrast, the GY, GN, and TDM traits showed more dispersed sets, with little overlap between metrics and a predominance of associations specific to a single estimator. HI showed an intermediate pattern, where the Ratio and Linear metrics coincided with those derived from WW and WL, forming the group with the highest number of intersections between estimators. Overall, the plasticity methods revealed a heterogeneous distribution of signals, with a predominance of coincidences in developmental traits and less convergence in yield traits.

#### 2.4.3. Effect Sizes and Polygenic Architecture

The values obtained showed that, although the magnitude of the effects varied between plasticity metrics, in all cases the QTLs explained a small fraction of the total variance, generally below 10% (Table 3). This reflects the highly polygenic nature of the traits evaluated. It was observed that for GFP, MAT, and HI, the genetic proportion was higher than the environmental components, indicating greater genetic control in these traits. This pattern was repeated across the different plasticity metrics, especially in the phenotypic means, where VG values reached 8.4% for GFP, 10.4% for MAT, and 11.8% for HI, contrasting with VE, which were close to zero in most cases. In contrast, GY and GN showed a high contribution of residual error, suggesting a predominant influence of external factors and interactions not captured by the models. These results are consistent with high environmental sensitivity to water stress. For TDM, the different models did not detect consistent QTLs, confirming the weak genetic signal in this trait or a much more polygenic structure. In contrast, HI showed the highest VG values, especially in the phenotypic means (G = 11.78%) and in the Ratio (8.31%). Overall, comparisons between metrics showed that phenotypic means explained a greater proportion of the variance than phenotypic plasticity (RDPI, Ratio, and Linear; explanatory values below 10%). Likewise, in very few cases was the environmental component significant, suggesting that differences between genotypes and genotype × environment interaction dominate.

### 2.5. Functional Annotation and GO Enrichment and Genomic Distribution of Significant SNPs

#### 2.5.1. Genomic Distribution of Significant SNPs

**Figure 4:**
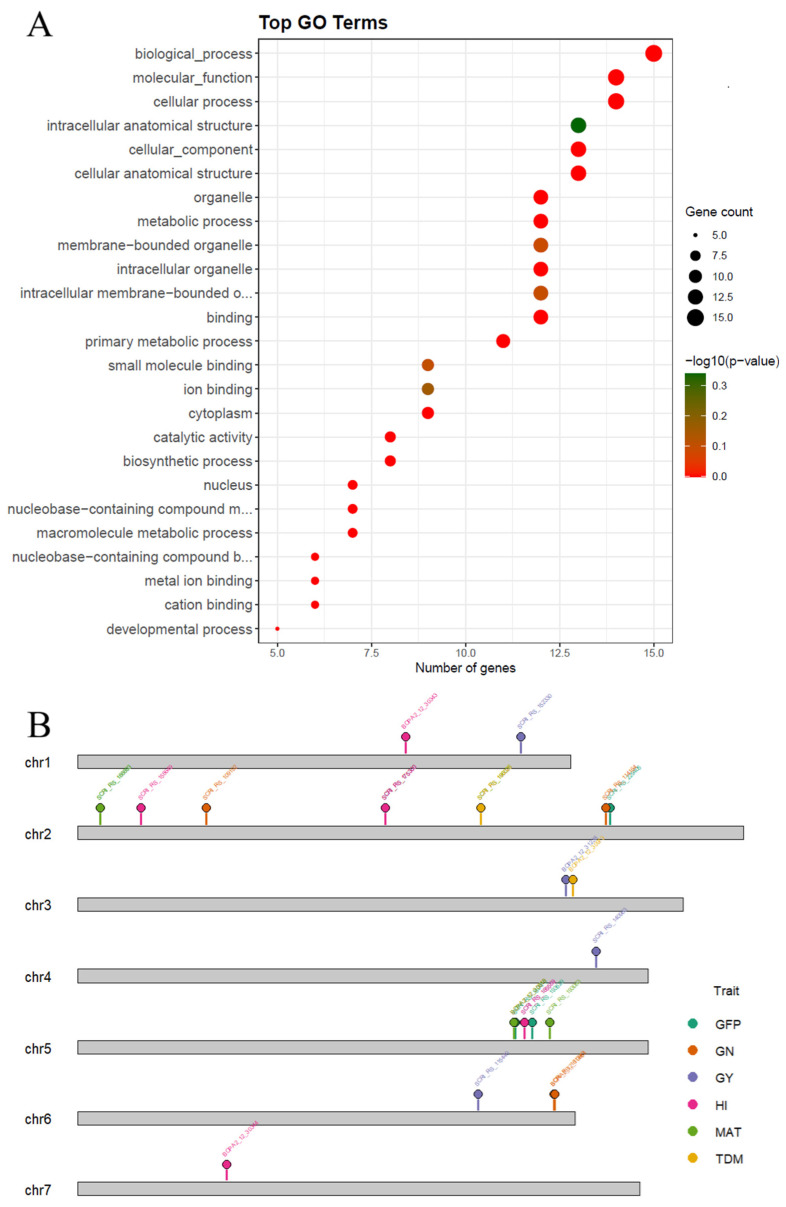
Functional characterization of significant markers located in coding regions detected in the GWAS for water tolerance in barley. (**A**) Associated genes were annotated, GO terms identified, and pathway enrichment visualized. (**B**) Although the reduced number of markers limited resolution for genetic mapping, several significant terms were identified, supporting biological relevance.

Functional annotation of the retained SNPs showed that a significant proportion were located in coding regions of the genome, indicating that several of the detected markers could have direct effects on functional genes or gene regions. GO term enrichment analyses identified a prominent representation of metabolic process, catalytic activity, and ion binding. These categories group genes associated with primary metabolic processes, enzymatic activity, and cellular regulation, consistent with the plant’s response to water deficit conditions. Most of the annotated genes were concentrated in cellular and metabolic processes (Figure 4), including biosynthetic process, binding, and macromolecule metabolic process, which had the highest number of genes and moderate statistical significance values. The presence of terms such as organelle and intracellular membrane-bound organelle indicates that some of the associated genes participate in cell organization and organelle structure, while others that are less represented are associated with cation and metal ion bindings and even with developmental processes. Overall, the enrichment results suggest that the genomic regions detected are linked to basic cellular functions and metabolic maintenance during exposure to water stress.

The candidate gene annotation (Table 4) efficiently complemented this analysis by identifying SNPs located in genes with known functions in signaling, metabolism, and growth regulation. Among them were proteins with HEAT repeats, hyperosmolarity-dependent calcium channels, serine/threonine kinases, sucrose phosphate synthase, gibberellin receptors, sucrose phosphate synthases, and enzymes involved in energy metabolism. These genes were mainly associated with the GFP, GY, HI, GN, and MAT traits, and several of them were present for the plasticity metrics and in more than one GWAS methodology. The chromosomes with the most relevant associations were 2H, 3H, 4H, 6H, and 7H. Together, functional annotation and GO enrichment confirm that the identified SNPs are grouped into relevant metabolic and structural pathways linked to the response to contrasting water availability conditions.

The candidate genes listed in Table 4 represent loci prioritized based on consistent detection across multiple GWAS methodologies and plasticity estimators. While experimental validation of gene expression or function was beyond the scope of this study, the integration of genomic position, functional annotation, and pathway-level enrichment provides a robust framework for biological interpretation of plasticity-associated loci to the water stress.

The chromosomal distribution of significant coding SNPs is illustrated in the kayogram (Figure 4B). This visualization showed the physical positions of the associated loci on the seven barley chromosomes, with color codes distinguishing the implicated traits. A non-random distribution of SNPs was observed, with clear clustering in certain chromosomal regions, particularly on chromosomes 2H and 5H, suggesting potential hotspots of trait-associated variation. Some markers shared across traits were observed on the same chromosomes. These localized concentrations of markers could indicate linkage disequilibrium with functional genes or regulatory regions involved in stress tolerance and metabolic regulation. SNP positions and gene annotations were defined based on the *Hordeum vulgare* cv. Morex reference genome (Ensembl Plants, assembly IBSC v2), using the corresponding annotation release.

#### 2.5.2. Allele-Specific Plasticity Patterns

**Figure 5:**
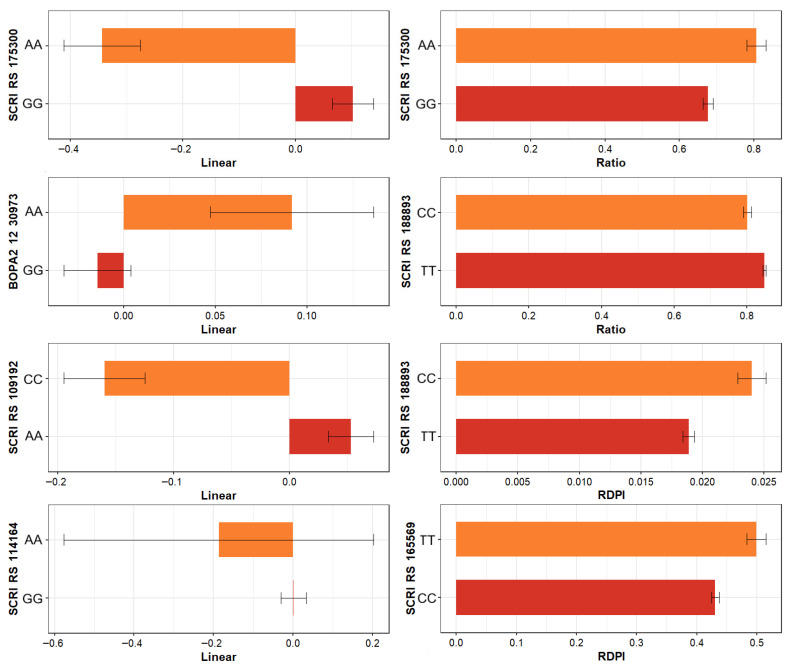
Representative examples of eight SNPs (each subpanel) located in coding regions associated with plasticity indices (i.e., linear and RDPI, respectively, in the left and right column of subpanels). For these markers, phenotypic plasticity varied between genotypes (*y* axes) in opposite directions (*x* axes) suggesting antagonistic pleiotropy, highlighting the complex interaction between genetic variants, metabolic pathways, and trait responses under water-limited condition.

SNPs located in coding regions were plotted together with the phenotypic plasticity values corresponding to their genotypes (Figure 5). A recurring pattern was observed, where the direction of the response varied between alleles of the same marker. Some genotypes carrying a certain allele showed increases under WL conditions, while others showed reductions in the same trait. This variation in response between alleles within the same locus demonstrates genetic heterogeneity in the expression of plasticity and reflects that the effects associated with each marker can manifest in opposite ways depending on the genetic and environmental context.

## 3. Discussion

Water limitation remains one of the most critical constraints on barley productivity worldwide [39,40,41,42], and understanding the genetic mechanisms that shape plant responses to fluctuating hydrological conditions is essential for climate-resilient agriculture [4,10]. Here we used a large and genetically diverse barley panel [8] to explore the genomic architecture of phenotypic plasticity under contrasting water regimes [20]. After integrating multiple plasticity estimators and four complementary GWAS approaches, we were able to provide one of the most comprehensive assessments to date of the loci and biological pathways underlying plastic responses to drought, and test its context-dependent effects.

### 3.1. Plasticity Is Trait-Dependent and Genotype-Specific

Phenotypic analyses revealed that water limitation caused substantial reductions in yield and biomass traits, whereas phenological traits were comparatively stable. These contrasting patterns reflect differences in the sensitivity of developmental versus reproductive processes to drought [33], consistent with previous reports in barley and other cereals [15,39,42]. Importantly, reaction norm analyses showed that plasticity varied not only in magnitude but also in direction [43], some genotypes exhibited increases in certain traits under water stress.

This opposing behavior demonstrates that plasticity is not uniform [13], reinforcing that it constitutes a genetically regulated trait rather than a simple by-product of stress exposure [44]. The identification of genotypes showing improved performance under WL conditions suggests that different allelic polygenic combinations modulate growth-stress trade-offs [17,45], generating a spectrum of strategies ranging from conservative to opportunistic responses in the face of water scarcity [19,46]. Overall, these observations reinforce the conclusions of theoretical frameworks proposing that plasticity is multidimensional, genotype-dependent, and shaped by both environmental and genetic effects [17,18,47]. The variation observed here highlights the value of treating plasticity as an environment-dependent discrete phenotype that can be quantified and genetically dissected [19,48].

Variance decomposition revealed trait-specific contrasts in the relative importance of genetic, environmental, and residual components [31]. Plant developmental traits HEA and MAT displayed strong genetic control and relatively low environmental sensitivity, whereas yield-related traits were dominated by environmental effects, particularly water availability. The reduction in GW, TDM, and GY under drought, with increases in between-genotype variability, suggests that these traits depend more heavily on context-specific interactions and may be governed by many small-effect loci [8,49], consistent with their highly polygenic nature [18,50]. While residual variance was large for yield traits, genetic components were still detectable in several other traits (i.e., GFP, MAT, HI), enabling successful association mapping. The exclusion of traits dominated by residual noise (e.g., VDW, GW) improved the resolution of genomic and biological interpretations.

### 3.2. Combining Multiple Plasticity Estimators and GWAS Models Increases Detection Robustness

A key contribution of this study is the integration of diverse plasticity estimators [12], absolute values (WW and WL), Ratio, RDPI, and Linear slopes, to capture distinct facets of environmental sensitivity [11]. Although these metrics were positively correlated, each retained unique information, supporting the use of multiple estimators to avoid oversimplifying the plastic response [20,51]. Applying four GWAS methodologies spanning statistical (rrBLUP, BLINK) and machine learning approaches (RF, XGBoost) revealed both complementary and method-specific associations. Machine learning models identified a broader set of associations [52,53], likely capturing non-linear and interactive effects, while BLINK and XGBoost prioritized highly conservative signals. Importantly, SNPs were retained only when detected by at least two plasticity estimators, and methodological convergence (e.g., rrBLUP–RF or BLINK–rrBLUP intersections) further strengthened confidence in the identified loci. This consensus-based strategy reduces false positives inherent to single-method GWAS and enhances the reproducibility of detected QTLs, a major drawback of single-context GWAS mapping efforts [32,54,55].

The heterogeneity in overlap across traits is biologically informative [56]. Developmental traits (i.e., GFP and MAT) displayed high concordance among metrics and methods, reflecting more stable, genetically determined responses. In contrast, yield-related traits showed lower overlap, reinforcing their complex and environmentally sensitive nature. These patterns mirror observations from GWAS of drought plasticity in maize and barley [15,57], where developmental traits tend to be governed by more consistent genomic regions [16], while yield traits depend on diffuse, context-dependent genomic architectures.

### 3.3. Plasticity QTLs Are Polygenic, with Small-Effects, and Enriched in Coding Regions

Across traits and plasticity metrics, most QTLs explained small fractions of phenotypic variance (<10%), consistent with a highly polygenic architecture. This finding reinforces polygenic models of plasticity proposed in theoretical studies [45,58] and empirical results in crops such as maize, rice, and wheat [16,20]. The predominance of small-effect loci suggests that plasticity emerges from the cumulative action of many genes rather than a few major-effect regulators [15,59].

A notable outcome is the substantial proportion of significant SNPs located in coding regions [60]. This pattern suggests that putative functional variants within proteins, rather than only regulatory or intergenic polymorphisms [61,62], may play a prominent role in mediating responses to water availability [49]. Functional annotation and GO enrichment analyses support this interpretation. Enriched terms were dominated by categories related to primary metabolism, catalytic activity, intracellular processes, and ion binding. These functions align well with known drought-response pathways, including osmotic adjustment [63,64], ROS detoxification [65], carbohydrate remobilization, and cell homeostasis.

Candidate genes identified near significant SNPs further reinforce this biological context. We detected genes involved in signaling (e.g., serine/threonine kinases, calcium channels), metabolic reprogramming (e.g., sucrose phosphate synthase), growth regulation (e.g., gibberellin receptors), and structural processes (e.g., organelle organization). Many of these gene families have been implicated in drought tolerance and plastic developmental adjustments in other crops [40,42,66,67,68,69,70,71,72,73,74,75,76,77,78,79,80,81,82,83,84,85,86,87,88,89,90,91,92,93]. Their recurrence across traits and plasticity metrics highlights them as promising targets for functional validation.

Furthermore, chromosomal patterns reveal potential hotspots of plasticity regulation. The karyogram revealed clusters of significant SNPs on chromosomes 2H, 5H, 6H, and 7H. Some of these regions coincide with previously reported loci associated with drought tolerance, yield stability, and phenology, including the HsDry2.2 locus. These recurrent clusters may represent pleiotropy hotspots for plasticity regulation where multiple physiological pathways converge [94].

### 3.4. Opposite Allelic Responses Suggest Antagonistic Pleiotropy

The presence of shared loci across multiple traits and plasticity metrics suggests pleiotropy or tight linkage, reflecting the interconnected nature of physiological and metabolic adjustments under drought [95]. Plots linking allelic variants to plasticity metrics highlight another central finding, which is that alleles at the same locus can drive opposite phenotypic responses depending on the environment [32,47]. Some alleles increased trait values under drought, while others reduced them, supporting the hypothesis that plasticity arises from a complex interplay of additive, interactive, and potentially epistatic effects under an antagonistic pleiotropy framework [96]. This may suggest that drought-beneficial alleles may increase osmotic adjustment, reduce growth rate, or shift resource allocation, traits that are advantageous under stress but reduce yield or biomass under optimal conditions [15,18,97]. Substitution of the stress-beneficial alleles, in near-isogenic lines for instance [98,99], will cause a measurable performance penalty in non-stress conditions.

This allelic heterogeneity has relevant implications for plant breeding. For instance, it demonstrates that (i) plasticity cannot be treated as uniformly beneficial, (ii) loci underlying plasticity need to be evaluated for both direction and magnitude of response across contrasting contexts, and (iii) breeding for plasticity requires careful definition of desirable response trajectories, as different genotypes could converge to similar performance.

### 3.5. Perspectives

Our results reinforce the value of integrating multiple plasticity metrics and diverse GWAS methodologies to dissect the genetic basis of complex adaptive traits. Coding-region variants and metabolic regulators suggest that plasticity may be improved through targeted recurrent introgression or genomic selection strategies focused on alleles that optimize physiological flexibility under drought. Key implications for breeding pipelines include (i) the necessity to incorporate plasticity into selection frameworks (not only absolute trait values), (ii) QTLs with consistent signals across multiple plasticity estimators represent robust candidates for marker-assisted selection, and (iii) developmental plasticity (MAT, GFP) appears more genetically tractable via an assortment of GWAS methods and may serve as a stable predictor of adaptive responses to heterogeneous environments [93].

Additionally, yield plasticity is highly polygenic [48], suggesting genomic selection may be more effective than single-locus approaches [100]. Future work should envision functional validation of candidate genes, assessment of epistatic interactions, and analyses across broader environmental gradients trying to achieve the enviromics spectrum [57,101]. Integrating transcriptomic [102], metabolomic data [103] and pangenomic [104,105] would also help clarifying how these loci mechanistically modulate physiological pathways in response to stress [106,107,108].

## 4. Materials and Methods

### 4.1. Experimental Design

The experimental material consisted of a large collection of approximately 1277 barley genotypes (*Hordeum vulgare* L.), representing lines and families (25) covering a highly diverse range of genetic and geographical variation previously described in studies of genome-wide scan for the identification of flowering-independent effects [8].

Specifically, the panel corresponds to the HEB-25 population, a wild barley-derived nested association mapping population developed to capture broad genetic diversity relevant to drought adaptation and yield stability. This population was selected because it segregates for drought-responsive loci independently of flowering time and has been extensively validated for the study of genotype-by-environment interactions under water deficit conditions [8].

This diversity provided the appropriate basis for a detailed analysis of the genetic architecture of phenotypic plasticity, following the conceptual framework established by Valladares et al. [109,110] and Arenas et al. [111,112,113], who emphasized that plasticity is not a uniform trait, but rather a specific attribute of genotypes, dependent on genetic, environmental, and random factors [109,112].

The trials were conducted in two completely contrasting environments in terms of water availability, which has already been described in detail [8]. The well-watered (WW) environment represented an optimal growing scenario without water limitations. On the other hand, the water-limited (WL) environment was designed to induce water stress during critical stages of the cycle, particularly heading and grain filling.

Field trials were conducted over two consecutive growing seasons (2014–2015 and 2015–2016) under controlled conditions an insect-proof net house at the experimental farm of The Hebrew University of Jerusalem (Rehovot, Israel). Plants were grown in canals that allowed for differentiated irrigation between adjacent experimental units. To replicate the natural rainfall pattern of the eastern Mediterranean region, irrigation was applied in winter and gradually reduced until early spring. Water limitation was imposed quantitatively by reducing seasonal irrigation volumes (WW: 25–34 m^3^; WL: 13–26 m^3^, depending on the season). Drought stress intensity was monitored physiologically by measuring stomatal conductance at multiple developmental stages using a porometer, ensuring an approximate 30% reduction in conductance under WL compared to WW conditions [8].

The experimental design included completely randomized blocks with replicates in both environments, ensuring comparability and control of environmental variability. Throughout the cycle, highly relevant agronomic and developmental traits were measured: number of grains (GN), grain weight (GW), grain yield (GY), total biomass (TDM), harvest index (HI), days to heading (HEA), days to maturity (MAT), green biomass (GFP), and dry weight of stems (VDW). Phenological traits were recorded through field observations, while yield and biomass traits were quantified after harvest and drying in the laboratory. Genotypes were not classified a priori as drought-tolerant or drought-susceptible. Instead, tolerance-related responses were inferred a posteriori based on observed phenotypic measures and plasticity across contrasting water regimes, allowing an unbiased assessment of genotype-specific environmental response.

### 4.2. Calculation of Plasticity Metrics

Several complementary approaches were used to characterize phenotypes, each reflecting different dimensions of the response to the environment. First, we worked with absolute values under WW and WL, which represent the extremes of the response range. Additionally, we calculated metrics derived from phenotypic plasticity: the WW/WL ratio, which provides a simple relative measure of change, and the relative plasticity index (RDPI) [114,115], calculated as the absolute difference (or distance) between conditions divided by the sum of both, as follows (Equation (1)):

(1) $$RDPI=\;\left|{\;X}_{WW}-\;X_{WL}\;\right|\;/{\;X}_{WW}+\;X_{WL}$$

We also considered the coefficient of variation (CV) as a proxy for intra-genotype variability across environments and thus serve as an alternative indicator of plasticity, particularly when differences in scale or baseline values challenged ratio-based metrics [116]. However, due to its strong correlation with other estimators in our data (Figure S2) and to avoid redundancy, CV was excluded from the final association analyses.

Meanwhile, the linear estimator was obtained from the classic model of Finlay and Wilkinson (1963) [117]. The latter is based on the regression of the yield of each genotype against a gradient of environments, expressed as follows (Equation (2)):

(2) $$y_{ij}={µ}_{i}\;+\;b_{i}\;+\;E_{j}\;+\;\varepsilon_{ij}$$

where $y_{ij}$ corresponds to the phenotype of genotype *i* in environment *j*, ${µ}_{i}$ is the average of the genotype, $b_{i}$ is the slope that reflects its environmental sensitivity, and $\varepsilon_{ij}$ is the residual error. Genotypes with slopes close to one are interpreted as stable, while higher or lower values denote relative sensitivity or insensitivity, and it is the value of this slope that will represent linear plasticity [112,116]. Thus, the GWAS analysis was based on five main estimators: WW, WL, Ratio, RDPI, and Linear.

The differences between environments were evaluated using the nonparametric Wilcoxon signed-rank test, statistically confirming that the WL condition induced significant phenotypic variations. To decompose the variance of each trait, linear mixed models were used, in which the phenotype was expressed as follows (Equation (3)):

(3) $$y_{ij}={µ}_{i}\;+\;G_{i}\;+\;E_{j}\;+\;{\left(GE\right)}_{ij}\;+\;\varepsilon_{ijk}$$

where $G_{i}$ represented the genotypic effect, $E_{j}$ the environmental effect, ${\left(GE\right)}_{ij}$ the genotype-by-environment interaction, and $\varepsilon_{ijk}$ the residual error, as reported in Djabali et al. (2023) [118]. Using this framework, the proportions of variance attributable to genetics, environment, and residuals were estimated, quantifying the relative importance of each.

### 4.3. Genetic Mapping of Plastic Responses

Genomic analysis included genotyping using a dense panel of SNPs distributed across the seven chromosomes of barley. These were genotyped using the Infinium iSelect 9K barley chip [119], which consists of 7864 SNPs [120]. The inclusion of SNPs that were polymorphic in at least one HEB family and that met the predefined quality criteria [<10% missing data and no complete linkage disequilibrium with another SNP in the set] resulted in 5709 informative loci. Using these data, linkage disequilibrium decay (LD decay) was estimated, which will give us adequate resolution for genome-wide association mapping. Subsequently, the population structure was evaluated using principal component analysis (PCA), allowing us to detect and correct for the presence of substructures. In addition, a rapid reduction in association was observed as a function of physical distance.

All statistical analyses were performed using the R software environment (R version 4.4.2; [121]). Descriptive statistics, variance partitioning, and nonparametric tests were implemented using base R functions. Principal component analysis (PCA) was conducted using the prcomp() function. Genome-wide association analyses were performed using the corresponding software and packages associated with each method, while downstream data handling and integration were carried out in R. Data visualization and figure generation were performed using the ggplot2 package [122], ensuring consistent graphical representation across traits and plasticity estimators.

The GWAS was carried out using four independent methodologies representing two different paradigms. From the classical statistical approach, rrBLUP and BLINK were used. The rrBLUP analyses were performed in R using the rrBLUP package [123], which implements ridge regression best linear unbiased prediction models for genome-wide association studies. BLINK analyses were conducted using the GAPIT [124] framework. Default parameters were applied unless otherwise specified. The former is a ridge regression model that allows thousands of markers to be handled simultaneously, modeling the random effect of SNPs as u ~ N $\left(0,\;\sigma^{2}K\right)$, where K is the kinship matrix (or the already defined genetic structure). BLINK, on the other hand, is an extension of the classic FarmCPU model, incorporating linkage disequilibrium information and Bayesian selection criteria, increasing power and reducing false positives. In parallel, two machine learning methodologies were used: Random Forest (RF), which constructs multiple decision trees and combines their results, and XGBoost, a gradient-based boosting algorithm that optimizes a regularized loss function. To define reliable associations, SNPs were first identified independently within each GWAS method by trait and plasticity estimator. Only SNPs detected by at least two plasticity estimators were retained, and among these, loci identified by more than one GWAS methodology were prioritized as high-confidence associations. This conservative strategy was adopted to reduce false positives and ensure robustness across analytical frameworks.

### 4.4. Estimating the Relevance of Plasticity QTLs

The filtered SNPs were subjected to functional annotation using the barley reference genome in Ensembl Plants. Their location in coding, intronic, or intergenic regions was identified, and those in coding regions were selected for GO term enrichment analysis. The procedure included the construction of sets of genes associated with significant SNPs, the definition of a genome-wide reference set, and the application of overrepresentation tests (Fisher’s exact test) with false discovery rate (FDR) correction. Significantly enriched terms were organized into categories of biological processes, molecular functions, and cellular components. This annotation-based enrichment framework was specifically employed to provide a systematic, biologically grounded interpretation of the genetic signals derived from the GWAS, rather than to establish direct causal validation of individual loci. By assessing the collective functional patterns of SNP-linked genes, GO analysis enabled the identification of coherent biological pathways underlying phenotypic plasticity responses to drought, thereby reducing reliance on single-gene interpretations. Finally, associations between SNPs located in coding regions and plasticity values were plotted, visualizing how alternative alleles generated divergent phenotypic trajectories in different environments. Within the framework of this study, this integrative annotation-enrichment approach provides sufficient functional context to support the interpretation of plasticity-related QTLs without requiring further experimental validation.

## 5. Conclusions

This study demonstrates that drought-related phenotypic plasticity in barley is a genetically regulated and strongly trait-dependent phenomenon. Across a diverse panel of 1277 genotypes, we show that yield-related traits are predominantly shaped by environmental variation, while developmental traits maintain stronger genetic control. Integrating five complementary plasticity estimators with four GWAS methodologies enabled us to identify a robust set of loci consistently associated with plastic responses. These associated SNPs were enriched in coding regions and linked to pathways including osmotic regulation, carbohydrate metabolism, hormonal signaling, and ion transport, offering concrete genomic targets for improving drought resilience. Many alleles showed opposite effects across environments, suggesting that due to antagonistic pleiotropy plasticity cannot be captured by evaluating performance under a single condition. Instead, the identified loci highlight the need to breed for context-dependent responsiveness.

The strength of this study lies in the integration of multiple plasticity metrics with independent GWAS approaches, which increased robustness and reproducibility of genetic associations. By focusing on pathway-level functional enrichment rather than single-locus validation, the results provide a biologically coherent interpretation of plasticity-related loci. Overall, this framework supports the use of phenotypic plasticity as an explicit target in breeding strategies aimed at improving crop performance under variable water availability, although laboratory validation is still important.

## Figures and Tables

**Table 1:** Summary statistics of traits differing under contrasting water regimes. Descriptive statistics (mean, standard deviation, and standard error) of GFP values across environments (WW and WL). Values highlight reduced trait means under stress and increased variance across genotypes, consistent with differential plastic responses.

Trait	Trait Description	n	Well-Watered (WW)			Water-Limited (WL)		
			Mean	±	SE	Mean	±	SE
GFP	Grain-filling period	1279	36.01	±	0.16	30.09	±	0.14
GY	Grain yield	1279	18.33	±	0.11	7.63	±	0.14
TDM	Total dry matter	1279	59.93	±	0.31	42.17	±	0.26
MAT	Maturity	1279	136.8	±	0.16	132.3	±	0.16
GN	Grain number	1279	300.3	±	2.2	207.8	±	2.73
HI	Harvest index	1279	31.6	±	0.19	22.18	±	0.39

**Table 2:** Genetic (G), and water treatment (ENV) random effects within the trait variance.

Trait	Var. G	Prop. G	*p*-Value G	Var. ENV	Prop. ENV	*p*-Value ENV	Var. Residual	Prop. Residual
GFP	6.41	17.59	1.6 × 10^−35^	17.51	48.01	2.1 × 10^−259^	12.55	34.40
GN	2140.39	19.39	6.0 × 10^−30^	4254.48	38.53	1.5 × 10^−197^	4645.76	42.08
GW	0.00	0.82	0.25	0.00	75.48	0	0.00	23.70
GY	5.30	7.37	9.6 × 10^−41^	57.24	79.63	0	9.35	13.00
HEA	18.13	77.61	7 × 10^−289^	0.78	3.32	4.6 × 10^−52^	4.45	19.06
HI	19.00	13.78	2.9 × 10^−12^	43.54	31.59	2.7 × 10^−135^	75.29	54.62
MAT	8.99	29.22	2.7 × 10^−60^	10.14	32.95	1.3 × 10^−195^	11.64	37.83
TDM	30.89	12.86	2.0 × 10^−43^	158.72	66.09	0	50.55	21.05
VDW	21.22	28.36	2.1 × 10^−33^	12.94	12.29	3.0 × 10^−81^	40.68	59.35

**Table 3:** Genetic (G) and water treatment (ENV) random effects on trait variance. Proportion of variation captured by QTLs (%) from SNPs obtained across the four GWAS methods. Only SNPs present in at least two categories (WW, WL, Linear, Ratio, or RDPI) were retained. QTLs detected across multiple methodologies were compared for consistency. See trait abbreviation as in Table 1.

Trait	Plasticity Measure	G	E	Res. Error
GFP	Phenotypic means	8.41	4.63	1.92
	RDPI	2.84	0.02	2.65
	Ratio	1.75	0	5.23
	Linear	2.94	0	5.84
GY	Phenotypic means	5.41	0	2.07
	RDPI	1.05	0.01	2.57
	Ratio	0	0	2.95
	Linear	0	0	2.1
TDM	Phenotypic means	0	0	1.85
	RDPI	0	0	3.83
	Ratio	0	0	4.57
	Linear	0	0	3.45
MAT	Phenotypic means	10.4	0.056	0.34
	RDPI	0	0.07	2.32
	Ratio	0.83	0.06	4.55
	Linear	1.12	0.05	3.97
GN	Phenotypic means	2.52	0	2.63
	RDPI	0	0	2.11
	Ratio	0	0	3.61
	Linear	0	0	4.48
HI	Phenotypic means	11.78	0	4.06
	RDPI	1.28	0.003	1.97
	Ratio	8.31	0	5.67
	Linear	1.24	0.06	4.88

**Table 4:** Top candidate gene annotation identified by multiple GWAS approaches under water stress conditions in barley include loci involved in calcium signaling, stress response regulation, redox homeostasis, growth regulation, and carbohydrate metabolism, stressing key pathways underlying phenotypic plasticity.

Trait	Marker	Chr	Position	Gene_ID	Description
GFP	SCRI_RS_188893	2H	26,375,085	HORVU.MOR.9832	HEAT repeat-containing protein
GFP	SCRI_RS_175300	2H	3.56 × 10^8^	HORVU.MOR.4842	Hyperosmolality-gated Ca^2+^ permeable channel 3.1
GFP	SCRI_RS_196026	2H	4.66 × 10^8^	HORVU.MOR.1167	Similarly to H062205.9 protein (*Oryza sativa*)
GFP	SCRI_RS_190690	2H	5.26 × 10^8^	HORVU.MOR.9742	EDAT1-like protein (*Arabidopsis thaliana*)
GN	BOPA2_12_3061	5H	5.05 × 10^8^	HORVU.MOR.6703	Synaptotagmin-2 (*Arabidopsis thaliana*)
GN	SCRI_RS_161043	6H	5.51 × 10^8^	HORVU.MOR.3705	Nicotinate mononucleotide adenyltransferase
GN	SCRI_RS_163143	6H	5.51 × 10^8^	HORVU.MOR.8951	S-(hydroxymethyl)glutathione dehydrogenase
GY	SCRI_RS_175440	2H	3.56 × 10^8^	HORVU.MOR.4842	Hyperosmolality-gated Ca^2+^ permeable channel 3.1
HI	SCRI_RS_186949	4H	35937077	HORVU.MOR.3335	Serine/threonine-protein kinase homolog
HI	BOPA2_12_3034	7H	1.72 × 10^8^	HORVU.MOR.1424	Gibberellin receptor GID1L2
HI	BOPA2_12_3097	3H	5.72 × 10^8^	HORVU.MOR.5994	Sucrose phosphate synthase
MAT	SCRI_RS_188893	2H	26,375,085	HORVU.MOR.9832	HEAT repeat-containing protein

## Data Availability

The phenotypic plasticity metrics and all analysis scripts generated in this study will be made publicly available upon acceptance of the manuscript. The raw genomic data and the original phenotypic measurements used here are openly available from the publication “Genome scan identifies flowering-independent effects of barley HsDry2.2 locus on yield traits under water deficit” (Merchuk-Ovnat et al., 2018, *Journal of Experimental Botany*), https://doi.org/10.1093/jxb/ery016 [8].

## Supplementary Materials

The following supporting information can be downloaded at https://www.mdpi.com/article/10.3390/ijms27020652/s1.
