# Supplementary figures and images for: Unveiling the Genomic Architecture of Phenotypic Plasticity Using Multiple GWAS Approaches Under Contrasting Conditions of Water Availability: A Model for Barley
Source: Int J Mol Sci. 2026 Jan 8;27(2):652. doi: 10.3390/ijms27020652 (PMC12841421; doi:10.3390/ijms27020652)

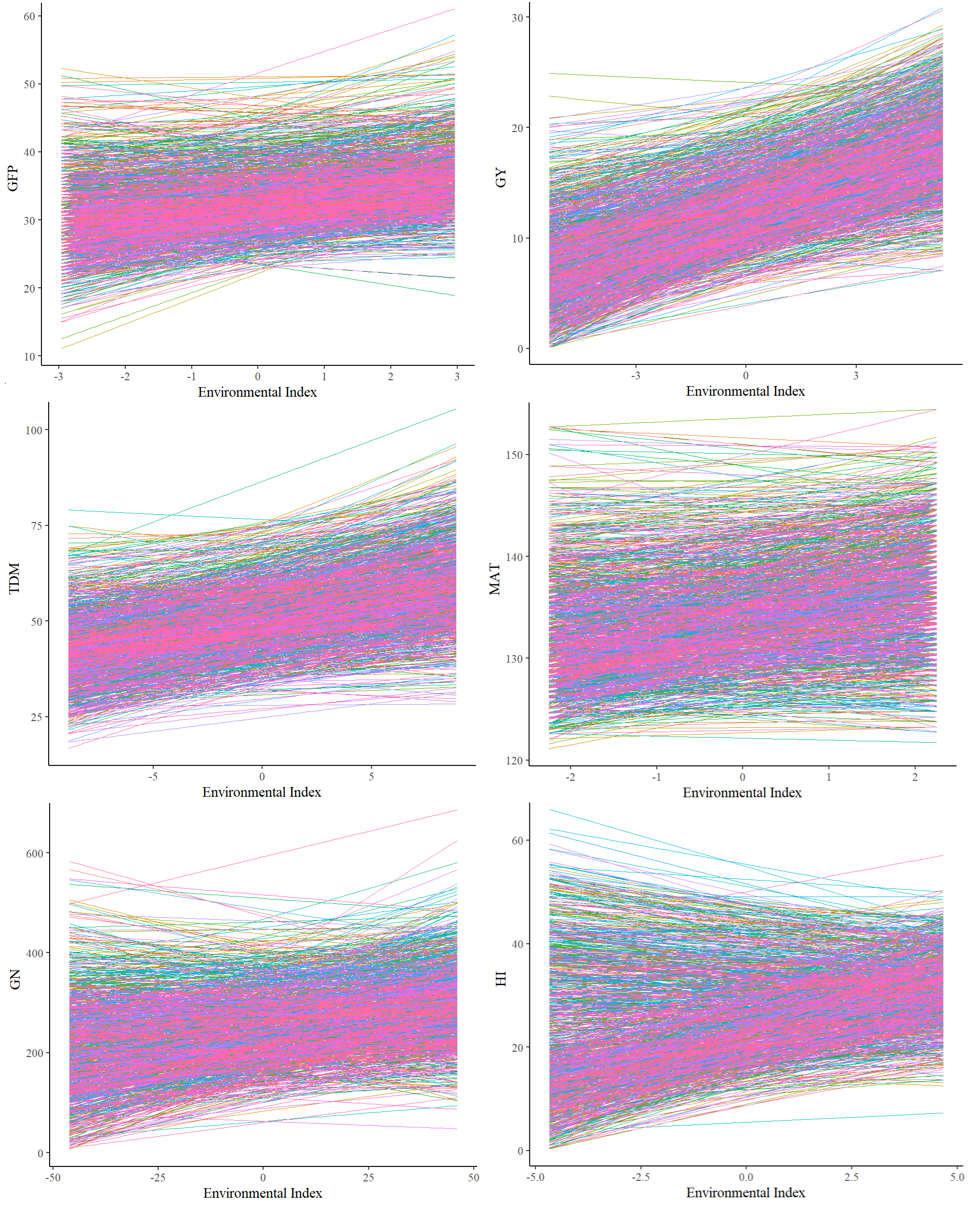

Supplement: Supplementary file 1 [file ijms-27-00652-s001.zip › FigureS1.tif]

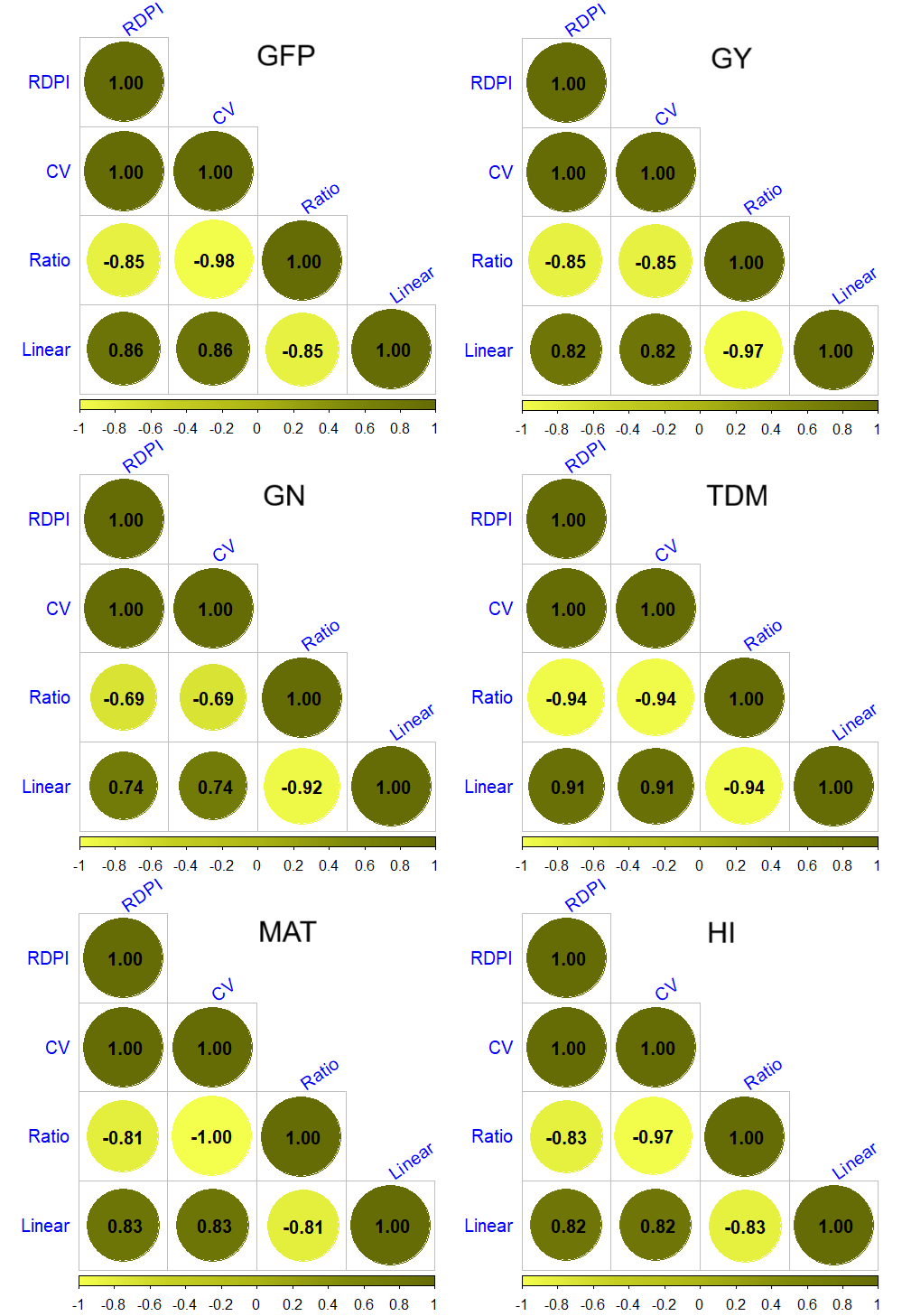

Supplement: Supplementary file 1 [file ijms-27-00652-s001.zip › FigureS2.tif]
